# Corylin accelerated wound healing through SIRT1 and PI3K/AKT signaling: a candidate remedy for chronic non-healing wounds

**DOI:** 10.3389/fphar.2023.1153810

**Published:** 2023-05-17

**Authors:** Yanghui Xiu, Yu Su, Lihua Gao, Hui Yuan, Sennan Xu, Ying Liu, Yan Qiu, Zhen Liu, Yuhang Li

**Affiliations:** ^1^ Eye Institute and Affiliated Xiamen Eye Center of Xiamen University, School of Medicine, Xiamen University, Xiamen, China; ^2^ Fujian Provincial Key Laboratory of Corneal and Ocular Surface Diseases, Xiamen, Fujian, China; ^3^ CAS Key Laboratory of Design and Assembly of Functional Nanostructures, Fujian Provincial Key Laboratory of Nanomaterials, Fujian Institute of Research on the Structure of Matter, Chinese Academy of Sciences, Xiamen, China; ^4^ Xiamen Institute of Rare-Earth Materials, Haixi Institutes, Chinese Academy of Sciences, Fujian, China

**Keywords:** corylin, phosphatidylinositol-3-kinase (PI3K), protein kinase B (AKT), sirtuin 1 (SIRT1), wound healing

## Abstract

**Introduction:** Chronic non-healing wound is a considerable clinical challenge and research into the discovery of novel pro-healing agents is underway as existing therapeutic approaches cannot sufficiently meet current needs.

**Method:** We studied the effects of corylin in cell line fibroblasts and macrophages by Western blots, PCR, Flow cytometry assay, Immunofluorescence.

**Results:** We showed that corylin, a main flavonoid extracted from *Psoralea corylifolia* L, reduced inflammatory responses, promoted collagen deposition, and accelerated the healing of full-thickness skin wounds in mice. Exploration of the underlying mechanisms showed that corylin activated the PI3K/AKT signaling, leading to fibroblasts’ migration, proliferation, and scratch healing. Corylin also activated sirtuin 1 (SIRT1) signaling, enhanced the deacetylation and cytoplasmic translocation of NF-κB p65, and therefore reduced lipopolysaccharide (LPS)-induced inflammatory responses in macrophages. Furthermore, inhibition of PI3K/AKT and sirtuin 1 pathway with LY294002 and EX527 prevent the therapeutic potency of corylin against chronic wounds.

**Conclusion:** In summary, our results suggested that corylin may be a candidate for the development of novel pro-healing agents.

## 1 Introduction

As the natural physical barrier to the external environment, the skin plays a crucial role in temperature regulation, as well as protection against microbial infection, and other environmental factors (Takeo et al., 2015). However, many insults, including accidental injury, surgery, burns, or metabolic dysfunction, can lead to skin damage, tissue loss, and structural and functional impairment (Han and Ceilley, 2017). The wounds healing after injury follow the processes of hemostasis, inflammation, proliferation, and remodeling (Kasuya and Tokura, 2014). Although wound healing is an innate and universal process, various factors, such as diabetes mellitus (DM), vascular disease, and aging, can affect the phases of healing, leading to chronic non-healing wounds (Dong et al., 2020). Chronic non-healing wounds increase rates of the invasion and infection of bacteria, resulting in sepsis, multiple organ failure, and death, as well as draining the medical system of an enormous number of resources (Martin and Nunan, 2015). Moreover, chronic non-healing wounds in the eye may result in opacification, perforation, discomfort, or visual loss (Williams et al., 2006). The inflammatory phase, which begins within hours after injury, is among the most relevant factors to delay wound healing. Excessive productions of pro-inflammatory cytokines including inducible nitric oxide synthase (iNOS), interleukin (IL)-1β, IL-6 and tumor necrosis factor-α (TNFα) are harmful to wound healing and can lead to chronic non-healing wounds (Martin and Nunan, 2015). Moreover, enhanced expressions of matrix metalloproteinases (MMP) in chronic wounds, promote the degradation of the local extracellular matrix (ECM), thus impairing cell migration in wounds (Tardaguila-Garcia et al., 2019). Fibroblasts are critical in the proliferation phase of wound healing and play a dominant role in collagen deposition, granulation tissue formation, and epithelialization (Liu et al., 2022). Currently, various therapeutic approaches have been used in the treatment of chronic non-healing wounds, including surgical debridement, antimicrobial agents, bioengineered skin equivalents, and growth factors (Han and Ceilley, 2017; Dubey et al., 2021; Hu et al., 2021; Sarojini et al., 2021; Wang et al., 2021). However, limited therapeutic effects and numerous side effects restrict their application (Han and Ceilley, 2017). New therapeutic approaches that could rapidly close the wound, while attenuating inflammation is still highly desired for the treatment of chronic non-healing wounds.

*Psoralea corylifolia* L. is a herb that has been widely used for the treatment of leucoderma and skin inflammatory-related diseases (Alam et al., 2018). Corylin is a main flavonoid that is extracted from *P. corylifolia* L (Alam et al., 2018). Corylin is known to improve atherosclerosis and ameliorate lipopolysaccharide (LPS)-induced inflammatory responses (Hung et al., 2017; Chen et al., 2020; Chen et al., 2021a). Corylin can promote osteoblast proliferation and differentiation through estrogen and Wnt/β-catenin signaling pathways (Yu et al., 2020). Corylin also reduces obesity and insulin resistance and promotes adipose tissue browning through sirtuin 1 (SIRT1) and β3-Adrenergic receptors (Chen et al., 2021b; Che et al., 2021). Additionally, treatment with corylin attenuates obesity-induced fatty liver disease, type 2 diabetes, and atherosclerosis by inhibiting heat shock protein 90β and promoting the degradation of mature sterol regulatory element-binding proteins (Zheng et al., 2019; Chen et al., 2020; Chen et al., 2021b). However, whether corylin could accelerate the healing of wounds remains unknown. The present study aimed to investigate the effects and the underlying mechanisms of action of corylin on wounds healing.

In this study, we examined the effects of corylin on wounds healing in mice. We showed that administration of corylin reduced inflammatory responses and accelerated healing of wounds *in vivo* and *in vitro*, supporting the idea that corylin may be considered a new treatment for chronic non-healing wounds.

## 2 Materials and methods

### 2.1 Chemicals

All reagents used in the present study were purchased from Sigma (Shanghai, China), seeking the highest grade commercially available unless otherwise indicated. To study the role of Sirt1, PI3K, and AKT in corylin-induced pharmacological actions, cells were treated with Sirt1 inhibitor EX527, ER antagonist Fulvestrant, PI3K inhibitor LY294002 and AKT inhibitor Miltefosine, respectively. EX527 (Cat. E129892), Fulvestrant (Cat. F125644), LY294002 (Cat. L408397), and Miltefosine (Cat. N130417) were purchased from Aladdin (Shanghai, China). Corylin was purchased from MedChemExpress (Cat. HY-N0236).

### 2.2 *In vivo* wound healing model

All experimental procedures and animal usage were carried out and approved by the Animal Care and Use Committee of Xiamen University. BALB/C male mice (18–22 g) were anesthetized with pentobarbital sodium and back cutaneous hair was removed by electrical shaving. One 10 mm diameter full-thickness skin wound was made on the dorsal surface with a round skin biopsy punch. For corylin treatment, mice (*n* = 6) received corylin (1, 3 mg in 0.1 mL acetone), EX-527 (0.5 mg), LY294002 (0.5 mg) or acetone vehicle control once daily for 8 days. The doses of corylin, LY294002 and EX527 were based on the preliminary experiments. The administration and route of corylin at the animal level were determined in the pharmacokinetic studies. The wounds were photographed, on day 0, day 2, 4, 6, and 8, using a digital camera. Wound areas (S) were measured by counting the total amount of grayscale pixels in the cell-free area with ImageJ software at 0 and 24 h. The migration rate (%) was calculated using the following formula: migration rate (%) = 1 - S (24 h)/S (0 h). FGF2 was used as the positive control. Where SW0 is the size of the initial wound area and SWn is the size of the wound area on day *n* post-surgery (Wang et al., 2021).

### 2.3 Histology

The dorsal skins of mice were harvested and fixed in paraformaldehyde at 4°C, followed by embedding in paraffin. Sections with a thickness of 5 μm were obtained by a microtome (Xie et al., 2022).

#### 2.3.1 Immunofluorescence staining

Paraffin-embedded dorsal skin sections were deparaffinized, rehydrated, and antigen was retrieved by incubating sections in boiling in sodium citrate buffer (10 mM, pH 6) for 15 min. Slides were then incubated with primary antibodies: rabbit anti-α-SMA (Abcam, Cat. ab5694, dilution 1:600) or rabbit anti-COL1A1 (Abcam, Cat. ab7778, dilution 1:600) at 4°C for 10 h. After incubation, sections were washed with 0.1 M PBS and incubated with goat anti-rabbit IgG-Alexa Fluor 488 (Abcam, Cat. ab150077, dilution 1:1000) or goat anti-rabbit IgG-Alexa Fluor 555 (Abcam, Cat. ab150078, dilution 1:1000) at room temperature for 1 h. After an additional rinse, sections were mounted by VECTASHIELD mounting medium with DAPI (Vector Lab, Shanghai, China) and observed under confocal microscopy (Olympus, Japan) (Li et al., 2021a). The fluorescence intensity was measured on fields (460 μm × 460 μm) around the wound area using the ImageJ software (Li et al., 2021a). At least six replicates were analyzed for each treatment.

### 2.4 Real-time quantitative PCR

Total RNA was extracted from skins and cell samples with Trizol (Invitrogen, Cat. 15596026) and measured by spectrophotometer (Beckman coulter, Shanghai, China) using a previously described method (Li et al., 2021b). cDNA was synthesized from 1 μg of total RNA by using the FastQuant RT Kit (TianGen, Cat. KR106-03) following the manufacturer’s instructions. Real-time quantitative PCR was performed in a 7300 real-time PCR System (Applied Biosystems, CA, United States) using SYBR Premix Ex Taq GC (Takara, Dalian, China). RNA levels were normalized using GADPH as an internal standard. The primer sequences for mouse genes were as follows:

α-SMA: 5’-ACT​GGG​ACG​ACA​TGG​AAA​AG-3’ (forward); 5’-CAT​CTC​CAG​AGT​CCA​GCA​CA-3’ (reverse).

TGF-β: 5’-TGA​TAC​GCC​TGA​GTG​GCT​GTC​T-3’ (forward); 5’-CAC​AAG​AGC​AGT​GAG​CGC​TGA​A) -3’ (reverse).

COL1A1: 5’-GAG​CGG​AGA​GTA​CTG​GAT​CG-3’ (forward); 5’-TAC​TCG​AAC​GGG​AAT​CCA​TC-3’ (reverse).

COL3A1: 5’-ATG​CCC​ACA​GCC​TTC​TAC​AC-3’ (forward); 5’-ACC​AGT​TGG​ACA​TGA​TTC​ACA​G -3’ (reverse).

Fibronectin: 5’-GAT​GTC​CGA​ACA​GCT​ATT​TAC​CA-3’ (forward); 5’-CCT​TGC​GAC​TTC​AGC​CAC​T-3’ (reverse).

IL-1β: 5’-TCG​CTC​AGG​GTC​ACA​AGA​AA-3’ (forward), 5’-CAT​CAG​AGG​CAA​GGA​GGA​AAA​C-3’ (reverse);

IL-6: 5’-ACA​AGT​CGG​AGG​CTT​AAT​TAC​ACA​T-3’ (forward), 5’-TTG​CCA​TTG​CAC​AAC​TCT​TTT​C-3’ (reverse);

iNOS: 5’-CCC​GTC​CAC​AGT​ATG​TGA​GGA​T-3’ (forward), 5’-CAT​TAC​CTA​GAG​CCG​CCA​GTG​A-3’ (reverse);

CCL-20: 5’-AAG​ACA​GAT​GGC​CGA​TGA​AG-3’ (forward), 5’-TCT​TGA​CTC​TTA​GGC​TGA​GGA-3’ (reverse);

TNF-α: 5’-TGA​ACT​TCG​GGG​TGA​TCG​GTC-3’ (forward), 5’-AGC​CTT​GTC​CCT​TGA​AGA​GGA​C-3’ (reverse);

GAPDH: 5’-AGT​GGC​AAA​GTG​GAG​ATT-3’ (forward); 5’-GTG​GAG​TCA​TAC​TGG​AAC​A-3’ (reverse).

### 2.5 Flow cytometry assay

Single-cell suspensions of skin samples were prepared using a previously reported method (Singh et al., 2016). Single-cell suspensions were incubated for 15 min at 4°C with FITC rat anti-mouse CD45 (BD Biosciences, Cat. 561088). After washing, the cells were assayed with a BD LSRFortessa X-20 flow cytometer according to the manufacturer’s protocol, and the data were analyzed using FlowJo software. Dead cells were excluded using the live/dead Hoechst33342 reagent (BD Biosciences, Cat. 561908). At least six replicates were analyzed for each treatment.

### 2.6 Cells culture

NIH/3T3 cells were cultured in DMEM supplemented with 10% FBS, 100 U/mL penicillin G and 0.1 g/mL streptomycin in humidified 5% CO_2_ atmosphere at 37°C, overnight until 50% confluence. Cells were then incubated with vehicle (0.1% DMSO), corylin (20 μM), EX-527 (1 μM), Fulvestrant (0.1 μM), LY294002 (10 μM) and Miltefosine (10 μM) for 24 h. Cells were then collected for Western blot and PCR studies (Qin et al., 2022). Raw264.7 cells were cultured in DMEM supplemented with 10% FBS and 2 mM glutamine at 37°C for 12 h until 80% confluence. Raw264.7 cells were then incubated with vehicle (0.1% DMSO), corylin (20 μM), EX-527 (1 μM), Fulvestrant (0.1 μM), LY294002 (10 μM) and Miltefosine (10 μM) for 30 min followed by treating with LPS (100 ng/mL, *Escherichia coli* 0111:B4, Sigma, Cat. L2630) for 24 h (Wu et al., 2019). Cells were then collected for further analysis. For immunofluorescence staining for p65, Raw264.7 cells were cultured on coverslips under the same conditions as described above. Immunofluorescence analysis was performed using previously reported methods (Li et al., 2021b). At least three replicates were analyzed for each treatment.

### 2.7 Cells proliferation assay

After culture at 37°C for 24 h, NIH/3T3 cells were then collected and incubated with MTT solution (Aladdin, D274386, 5 mg/mL, 10 μL) at 37°C for 4 h. The supernatant was then removed and DMSO (0.15 mL) was added. The reaction mixture was maintained at 37°C for 10 min, and continuously scanned at a wavelength of 490 nm for 3 min. Percent cell proliferation was defined as the relative percentage (%) of treated cells relative to the vehicle-treated control group (Wang et al., 2021). At least three replicates were analyzed for each treatment.

### 2.8 Western blots

Proteins were extracted from NIH/3T3 cells and Raw264.7 cells using RIPA lysis buffer and quantified by the BCA protein assay kit. Western blots were performed using the standard sodium dodecyl sulfate (SDS)–polyacrylamide gel electrophoresis method and enhanced chemiluminescence detection reagents (Thermo, WP20005) (Zhou et al., 2019). Primary antibodies against the following proteins were used: rabbit anti-mouse p-PI3K antibody (Abcam, Cat. ab182651, 1:1000 dilution), rabbit anti-mouse PI3K antibody (Abcam, Cat. ab86714, 1:1000 dilution), rabbit anti-mouse p-Akt antibody (Abcam, Cat. ab222489, 1:1000 dilution), rabbit anti-mouse Akt antibody (Abcam, Cat. ab8805, 1:1000 dilution), rabbit anti-mouse β-actin (Sigma, Cat. A5441, 1:50,000 dilution). As a secondary agent, the horseradish peroxidase-conjugated goat anti-rabbit secondary antibody (Cell Signaling Technology, 7074, dilution 1: 5,000) was used. Quantitative analyses were performed using ImageJ software, with β-actin as the internal standard.

### 2.9 Wound scratch assay

Wound scratch assays were performed in NIH/3T3 cells using a previously reported method (Wang et al., 2021). NIH/3T3 cells were seeded into 6-well plates and cultured in DMEM supplemented with 10% FBS, 100 U/mL penicillin G and 0.1 g/mL streptomycin in humidified 5% CO_2_ atmosphere at 37°C, overnight until 80% confluence. The medium was then replaced with serum-free DMEM containing mitomycin C (Aladdin, Cat. M407788, 15 μg/mL) and NIH/3T3 cells were cultured for 90 min to prevent cell proliferation. Scratch lines were made using a sterile pipette tip. Cells were then incubated with vehicle (0.1% DMSO), corylin (20 μM), EX-527 (1 μM), Fulvestrant (0.1 μM), LY294002 (10 μM) and Miltefosine (10 μM) for 36 h. Scratches lines were photographed 0–36 h after scratching. At least three replicates were analyzed for each treatment.

### 2.10 Statistical analysis

Data are presented as means ± SEM. Analyses were performed with either GraphPad Prism 9.0.5. Three or more different groups were analyzed by one-way ANOVA with Dunnett’s *post-hoc* multiple comparison tests. *p* < 0.05 was considered statistically significant.

## 3 Results

### 3.1 Topical application of corylin accelerated healing of full-thickness skin wounds in mice

**FIGURE 1:**
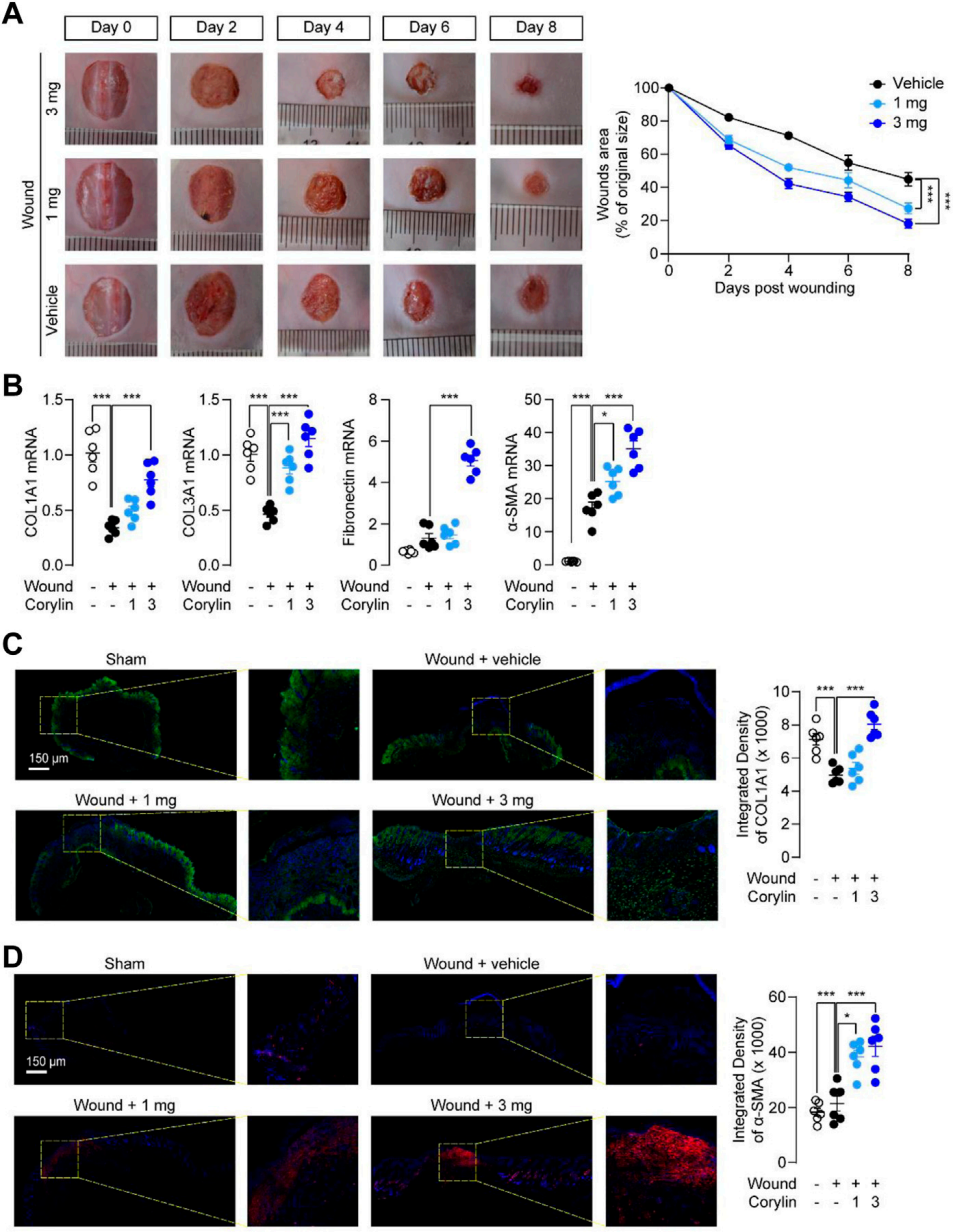
Topical application of corylin accelerated healing of full-thickness skin wounds in mice. **(A)** Representative images of skin wounds on days 0, 2, 4, 6, and 8 post-injuries in the vehicle, corylin (1 mg), and corylin (3 mg)-treated groups. Corylin was applied topically once daily to skin wounds. The amounts of wound healing at different time-points. Wound closure was measured by morphometric measurement of wound areas. **(B)** mRNA expressions of COL1A1, COL3A1, fibronectin and α-SMA in wound tissues at day 8. **(C)** Representative images of COL1A1 immunofluorescence staining at day 8 after corylin treatment. **(D)** Representative images of α-SMA immunofluorescence staining at day 8 after corylin treatment. *N* = 6 replicates per group, *p < 0.05; **p < 0.01; ***p < 0.001.

First, we examined whether corylin could promote the repair of full-thickness skin wounds in mice. Round wounds, penetrating the full-skin thickness of mice, were generated by a sharp punching pipe tool, and then treated once daily, either by a topical application of vehicle or by corylin for 8 days. As shown in Figures 1A, B, corylin treatment largely accelerated wound healing in mice. Fibroblasts are known as the source of collagen deposition during wounds healing, and they are the main cells expressing alpha-smooth muscle actin (α-SMA) (Martin and Nunan, 2015). Transforming growth factor (TGF)-β plays a critical role in wound healing by promoting the proliferation and migration of fibroblasts and collagen deposition (Martin and Nunan, 2015). Similarly, PCR analysis suggested that the mRNA expressions of COL1A1, COL3A1, fibronectin and α-SMA were increased when treated with corylin (Figure 1B). After determining the gross morphology of the wounds visually, we further investigated the effects of corylin on wound healing with COL1A1 collagen and α-SMA immunofluorescence staining. Compared with the vehicle control, corylin-treated wounded skin tissues showed higher intensity of COL1A1 deposition and α-SMA expression than the vehicle control (Figures 1C, D). These results suggested that corylin could promote wound healing and collagen deposition in mice.

### 3.2 Topical application of corylin ameliorated inflammatory responses in full-thickness skin wounds in mice

**FIGURE 2:**
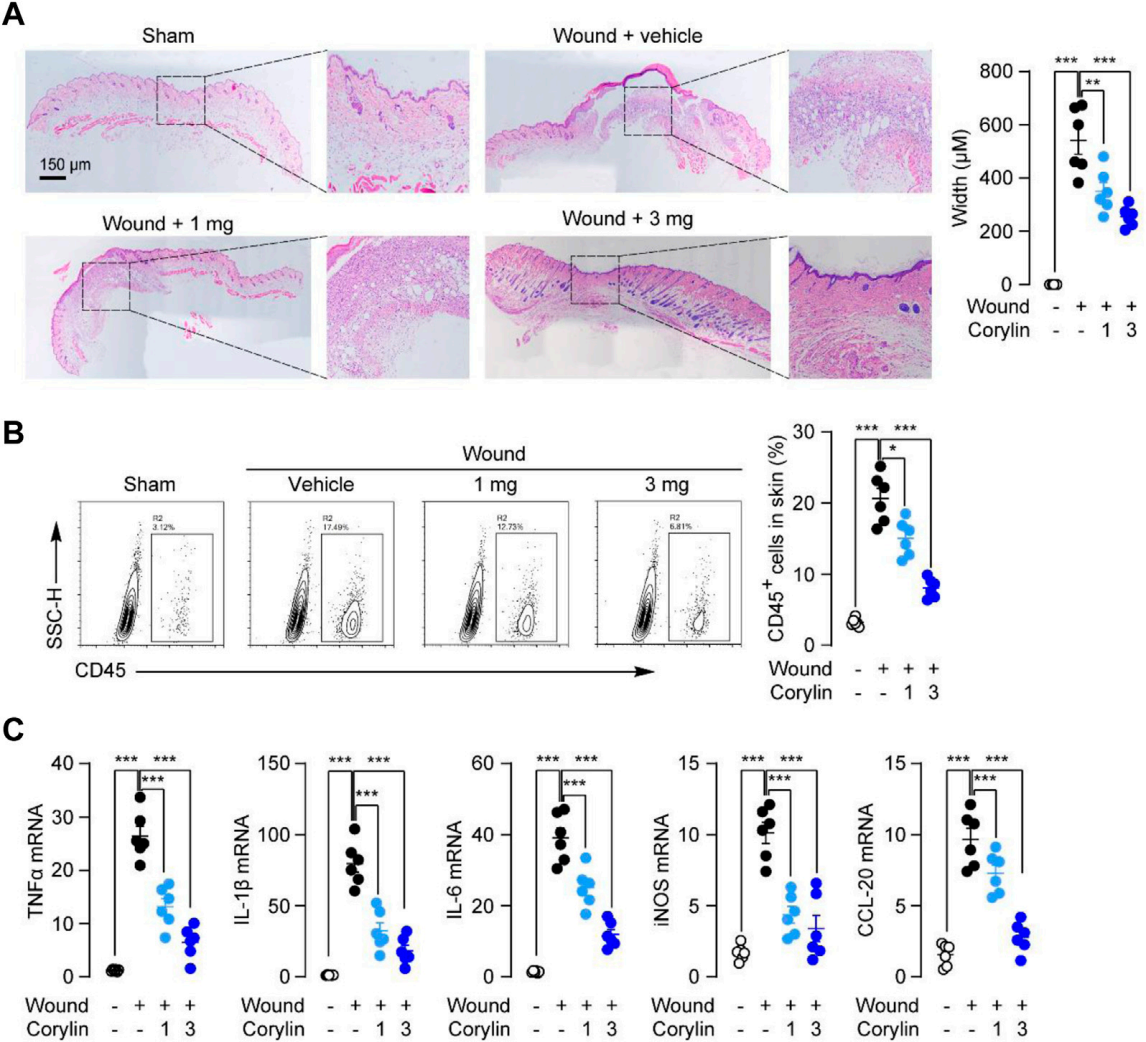
Topical application of corylin ameliorated inflammatory responses in full-thickness skin wounds in mice. **(A)** H&E staining of wound tissue on day 8 post-injuries in sham, vehicle, corylin (1 mg), and corylin (3 mg)-treated groups. Corylin was applied topically once daily to skin wounds. **(B)** Flow cytometry analysis of the frequency of CD45^+^ leukocytes in skin tissues. **(C)** mRNA expressions of TNF-α, IL-1β, IL-6, iNOS, and CCL-20 in wound tissues at day 8. *N* = 6 replicates per group, *p < 0.05; **p < 0.01; ***p < 0.001.

Next, we investigated the effects of corylin on inflammatory responses, an important factor that could delay wound healing (Takeo et al., 2015), in full-thickness skin wounds in mice. Histological analysis showed that fewer cellular and vascular components were observed in the granulation tissues of vehicle-control mice at day 8, while corylin treatment increased cells and blood vessels in the granulation tissues in mice (Figure 2A). In addition, flow cytometry also demonstrated that corylin suppressed the infiltration of CD45^+^ leukocytes into wounded skin tissues (Figure 2B). Furthermore, tissue injury elicited a drastic increase in the mRNA expressions of pro-inflammatory factors, including TNFα, IL-1β, IL-6, iNOS, and CCL-20 in the wounded skin tissues, while corylin significantly inhibited the increment of these cytokines (Figure 2C). These data suggested that treatment with corylin could ameliorate inflammatory responses in full-thickness skin wounds in mice.

### 3.3 Corylin promoted migration, proliferation, and scratch healing of NIH/3T3 fibroblasts through the PI3K/AKT pathway

**FIGURE 3:**
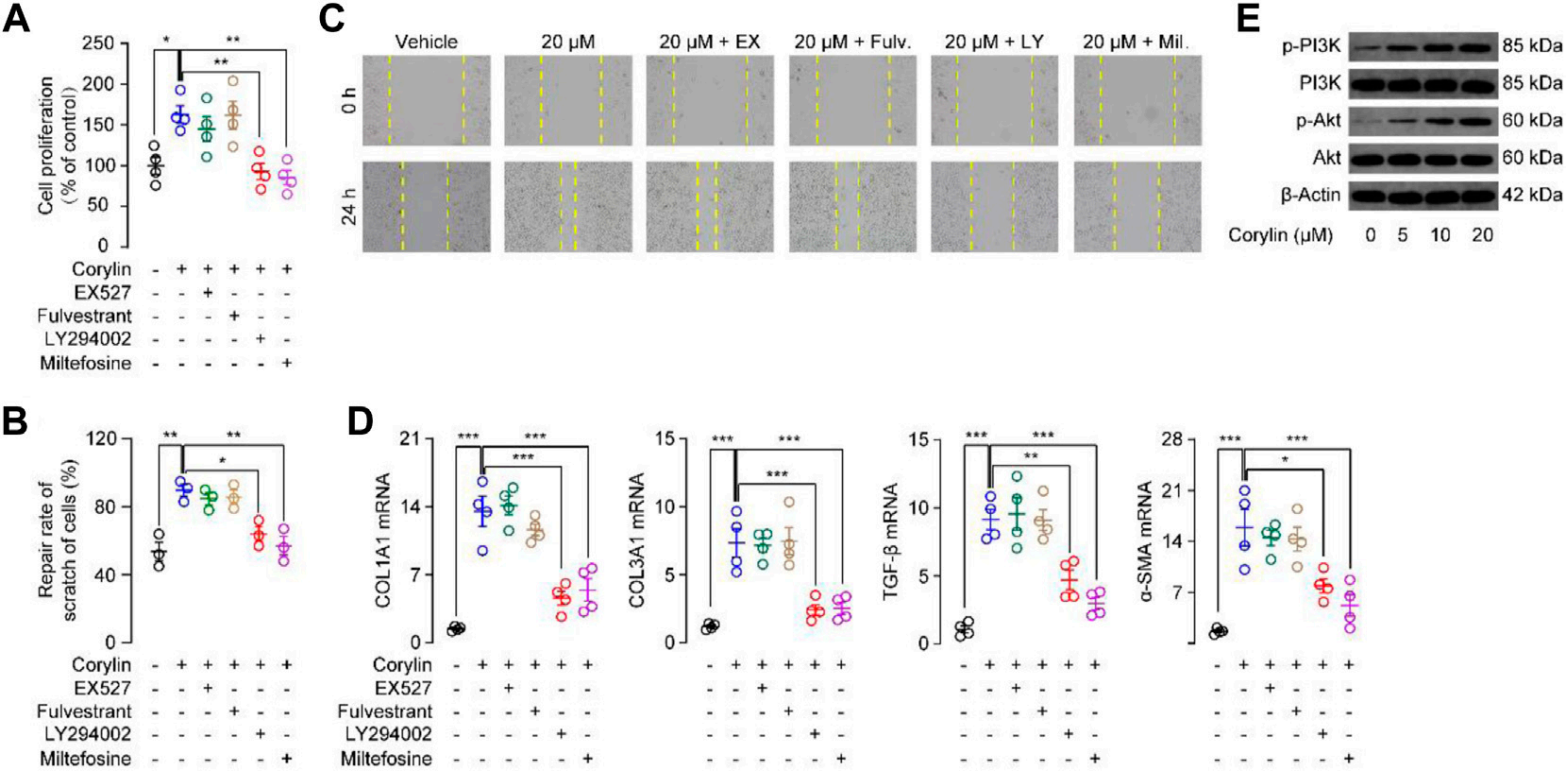
Corylin promoted migration, proliferation, and scratch healing of NIH/3T3 fibroblasts through the PI3K/Akt pathway **(A)** Proliferation of NIH/3T3 fibroblasts was assessed by MTT assays. **(B)** Quantitative analysis of migration rate in NIH/3T3 cells after incubation with corylin over 24 h. **(C)** Representative images displaying effects of vehicle (0.1% DMSO), corylin (20 μM), EX-527 (1 μM), fulvestrant (0.1 μM), LY294002 (10 μM) and miltefosine (10 μM) on the healing of NIH/3T3 cells scratches **(D)** mRNA expressions of COL1A1, COL3A1, TGF-β and α-SMA in NIH/3T3 fibroblasts after incubation with corylin over 24 h. **(E)** Representative Western blot images displaying the effects of corylin on expression and phosphorylation levels of PI3K and Akt. *N* = 3-4 replicates per group, *p < 0.05; **p < 0.01; ***p < 0.001.

The proliferation of fibroblasts is a critical process during wound healing (Martin and Nunan, 2015). We then tested whether corylin could affect the proliferation of fibroblasts by the MTT method. First, we gave various doses of corylin (5–80 μM) and analyzed the proliferation of NIH/3T3 fibroblasts at 12 and 24 h. MTT assay showed that corylin dose-dependently enhanced the number of live NIH/3T3 fibroblasts, compared to the vehicle control group (Supplementary Figure S1A). However, high doses of corylin (100–800 μM) showed cytotoxic effects in NIH/3T3 fibroblasts (Supplementary Figure S1A). Thus, corylin was administrated at a dose of 20 μM for 24 h. Previous studies showed that PI3K and AKT signaling proteins are involved in the wound healing process (Castilho et al., 2013; Martin and Nunan, 2015). Moreover, corylin also affects SIRT1 and estrogen receptor (ER) signaling (Yu et al., 2020; Chen et al., 2021b). Therefore, we further tested whether PI3K, AKT, SIRT1, and ER signaling participate in the proliferation of fibroblasts. We observed that treatment with PI3K inhibitor LY294002 and AKT inhibitor Miltefosine, but not SIRT1 inhibitor EX-527 or ER antagonist Fulvestrant, significantly reduced the proliferation of fibroblasts (Figure 3A), indicating that PI3K/AKT pathway may involve in corylin-induced fibroblasts proliferation. We also did the cell cycle analysis with EdU assay. As shown in Supplementary Figure S2, EdU assay results exhibited that corylin facilitated the proliferation of NIH/3T3 cells. Treatment with LY294002 and miltefosine, but not EX-527 or fulvestrant, significantly reduced the corylin-induced proliferation of NIH/3T3 cells. The migration of fibroblasts to injured sites is an important process for wound healing. Scratch (wound) assay showed that corylin could induce the migration of NIH/3T3 fibroblasts to scratch, resulting in wound closure (Figures 3B, C). The migratory effects of corylin on fibroblasts seem to be mediated by PI3K/AKT pathway, as LY294002 and Miltefosine could suppress such actions (Figures 3B, C). In addition, we further examined the effects of corylin on the activation of fibroblasts. We found that the mRNA expressions of COL1A1, COL3A1, α-SMA, and TGF-β were dramatically higher in corylin-treated fibroblasts than in the vehicle control group. Such effects were blocked by LY294002 and Miltefosine, but not EX-527 or Fulvestrant (Figure 3D). Furthermore, we asked whether corylin affects the functions of PI3K/AKT signaling. Indeed, Western blot results suggested that corylin dose-dependently increased the phosphorylation of PI3K and AKT (Figure 3E). Taken together, these data suggested that corylin promoted proliferation, migration, and scratch healing of NIH/3T3 fibroblasts through the PI3K/AKT pathway.

### 3.4 Corylin reduced LPS-induced inflammatory responses in Raw264.7 cells through the SIRT1/NF-κB pathway

**FIGURE 4:**
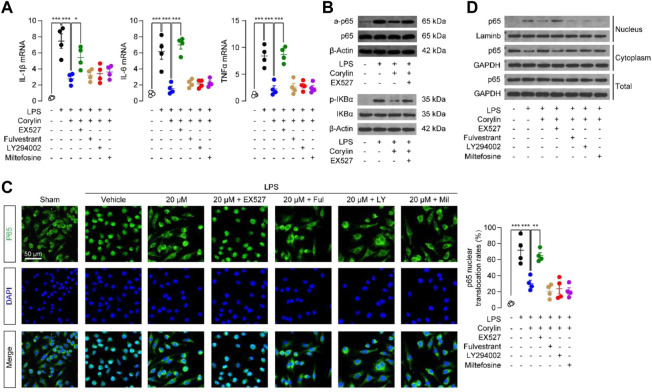
Corylin reduced LPS-induced inflammatory responses in Raw264.7 cells through the SIRT1/NF-κB pathway **(A)** Effects of vehicle (0.1% DMSO), corylin (20 μM), EX-527 (1 μM), fulvestrant (0.1 μM), LY294002 (10 μM) and miltefosine (10 μM) on mRNA expressions of TNFα, IL-1β and IL-6 in Raw264.7 cells after treatment with LPS for 24 h. **(B)** Representative Western blot bands of the acetylated p65 (a-p65) abundances in Raw264.7 cells. **(C)** Representative confocal images and quantification of the nuclear translocated p65 in Raw264.7 macrophages. **(D)** Representative Western blot bands of p65 abundances in the cytoplasmic and nuclear fractions of Raw264.7 cells. *N* = 4 replicates per group, *p < 0.05; **p < 0.01; ***p < 0.001.

Macrophages play an important role during wound healing (Martin and Nunan, 2015). They take up and clear dead cells, release proteases, and produce chemotactic chemokines/cytokines (Martin and Nunan, 2015). Although these are critical actions to set the stage for tissue repair, excessive or prolonged inflammation may delay the healing of wounds. Thus, we tested whether corylin could reduce inflammatory responses in Raw264.7 macrophages. As shown in Figure 4A, LPS stimulation of Raw264.7 cells increased mRNA expression of pro-inflammatory cytokines, including IL-1β, IL-6, and TNF-α, while these inductions could be suppressed by corylin. Furthermore, the anti-inflammatory effects of corylin were blocked by SIRT1 inhibitor EX527 (Figure 4A), suggesting that corylin may alleviate inflammation through a SIRT1 activation mechanism. NF-κB is an important family of transcription factors that is responsible for the inflammatory actions in macrophages. We then tested whether corylin could affect NF-κB signaling. Western blot results showed that LPS induced the acetylation of NF-κB p65, while corylin could reduce such actions (Figure 4B). Additionally, treatment with EX527 could restore decreased levels of acetylated p65 induced by corylin (Figure 4B).

We examined the protein expressions of p-IκBα in Raw264.7 cells after treatment with LPS and corylin for 24 h. As shown in Figure 4B, LPS induced the phosphorylation of IκBα, while corylin could reduce such actions. Additionally, treatment with EX527 could restore the decreased levels of p-IκBα induced by corylin. Moreover, we also investigated the effects of corylin on the nuclear translocation of p65. Treatment with LPS enhanced the nuclear translocation of p65 in Raw264.7 cells, while corylin significantly suppressed such actions (Figure 4C). SIRT1 inhibitor EX527 prevented the effects of corylin on the p65 nuclear translocation in macrophages (Figure 4C). We also extracted cytoplasmic and nuclear fractions and examined the protein levels of p65 in these fractions. Similar to the results of the immunofluorescence assay, corylin treatment reduced the protein levels of p65 in nuclear fractions, while EX527 could restore the increased levels of nuclear p-p65 induced by LPS (Figure 4D). When combined, these results suggested that corylin reduced inflammation through the SIRT1/NF-κB pathway.

### 3.5 Corylin accelerated healing of full-thickness skin wounds in mice through the PI3K/Akt pathway and SIRT1/NF-κB pathway

**FIGURE 5:**
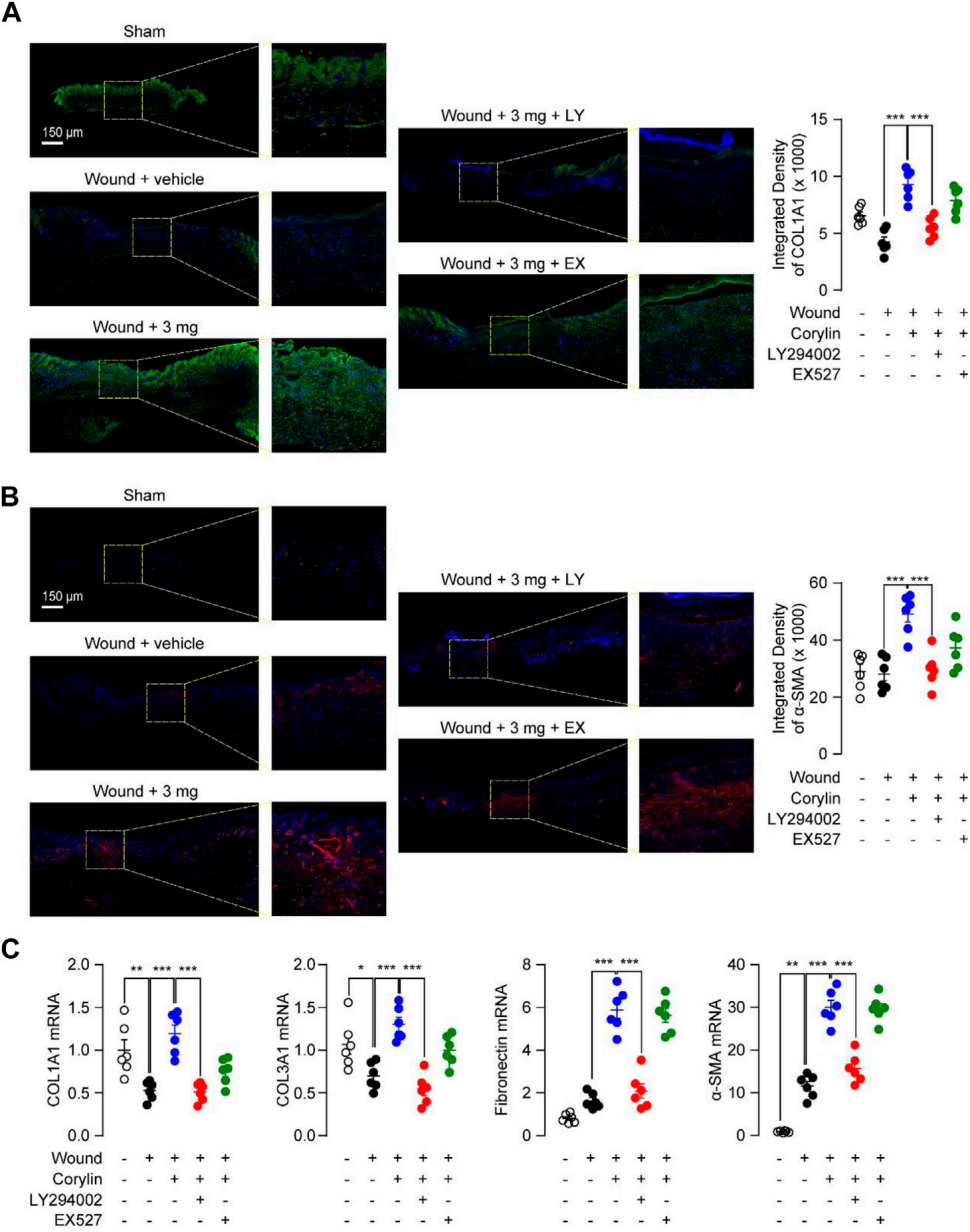
Corylin accelerated the healing of full-thickness skin wounds in mice through the PI3K/Akt pathway. Representative immunofluorescence images of **(A)** COL1A1 and **(B)** α-SMA at day 8 post-injuries in sham, vehicle, corylin (3 mg), EX-527 (0.5 mg) and LY294002 (0.5 mg)-treated groups. Compounds were applied topically once daily to skin wounds. **(C)** mRNA expressions of COL1A1, COL3A1, fibronectin and α-SMA in wound tissues at day 8. *N* = 5-6 replicates per group, *p < 0.05; **p < 0.01; ***p < 0.001.

Encouraged by the *in vitro* mechanism studies, we further confirmed the signal pathways underlying the effects of corylin *in vivo*. PI3K inhibitor LY294002 and SIRT1 inhibitor EX527 were topically applicated to wound tissues in corylin-treated mice. Corylin-induced collagen deposition and α-SMA expression during wounds healing were significantly suppressed by LY294002 (Figures 5A, B). In addition, inhibition of PI3K activity by LY294002 significantly reduced the mRNA levels of COL1A1, COL3A1, fibronectin and α-SMA in corylin-treated wounded skin tissues (Figure 5C). Although EX527 could partially block the pharmacological effects of corylin in mice, it had poor effects on the production and deposition of collagen, as well as mRNA expressions of α-SMA in corylin-treated wounded skin tissues (Figures 5A–C).

**FIGURE 6:**
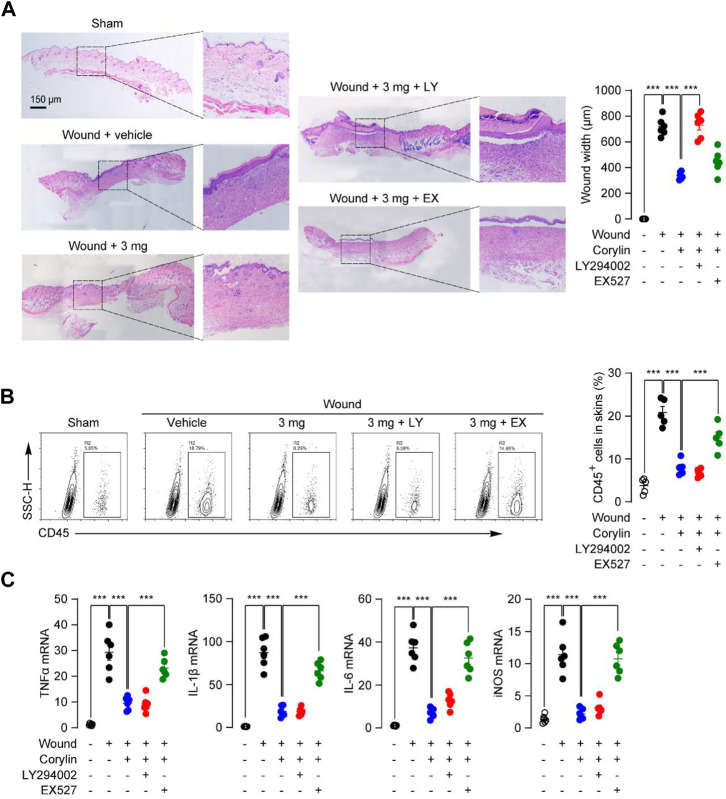
Corylin ameliorated inflammatory responses in full-thickness skin wounds in mice through the SIRT1/NF-κB pathway. **(A)** Representative H&E staining images of wound tissue on day 8 post-injuries in sham, vehicle, corylin (3 mg), EX-527 (0.5 mg) and LY294002 (0.5 mg)-treated groups. Compounds were applied topically once daily to skin wounds. **(B)** Flow cytometry analysis of the frequency of CD45^+^ leukocytes in skin tissues. **(C)** mRNA expressions of TNF-α, IL-1β, IL-6, iNOS, and CCL-20 in wound tissues at day 8. *N* = 6 replicates per group, *p < 0.05; **p < 0.01; ***p < 0.001.

We also examined whether PI3K and SIRT1 signaling participated in the anti-inflammation actions of corylin in mice. Inhibition of SIRT1 activity by EX527 reversed the protective effect of corylin against leukocyte infiltration in skin-wounded tissues, as demonstrated by H&E staining and flow cytometric analysis (Figures 6A, B). Similar results were observed in PCR analysis, EX527 effectively prevents the anti-inflammatory actions of corylin in wounded tissues in mice (Figure 6C). However, LY294002 had no such effects (Figures 6A–C). These data further confirmed the role of the PI3K/Akt pathway and SIRT1/NF-κB pathway in the corylin-mediated promotion of wounds healing.

## 4 Discussion

Chronic non-healing wound is a serious clinical problem and research into finding effective wound healing strategies is underway there is no ideal treatment due to the complexity of the healing process (Kasuya and Tokura, 2014). In the present study, we showed that corylin could accelerate the healing of full-thickness skin wounds in mice. We also found that corylin promoted migration, proliferation, and scratch healing of fibroblasts, and reduced LPS-induced inflammatory responses in macrophages. The mechanism of corylin enhancing wound healing may be related to the activation of the PI3K/AKT and SIRT1 signaling. These results suggested that corylin may be a new therapeutic agent for chronic wounds healing.

Healing of wounds is a dynamic and complex process that develops through 4 typical stages: hemostasis, inflammation, proliferation, and tissue remodeling with scar formation (Takeo et al., 2015). Inflammation in the wound is generally caused by resident and infiltrated immune cells, including monocytes/macrophages, neutrophils, and T cells (Martin and Nunan, 2015). Our results showed that corylin effectively attenuated CD45^+^ immune cells’ recruitment into wounded tissues (Figure 2B). Moreover, corylin suppressed expressions of inflammatory cytokines or chemokines in wound tissues (Figure 2C). These anti-inflammatory actions of corylin seem to be mediated by SIRT1, as inhibition of SIRT1 activity could prevent the anti-inflammatory effects of corylin (Figures 6A–C). Macrophages play an important role in the inflammation stage of wound healing (Figure 4A). They also produce many cytokines associated with wound healing, including IL-1β, TNF-α, IL-6, and iNOS (Martin and Nunan, 2015). Our data suggested that corylin could suppress the acetylation and nuclear translocation of NF-κB p65 through a SIRT1-dependent way, and prevent inflammatory factors production in macrophages, thus enhancing wounds healing (Figures 4A–C).

Moreover, we found that corylin could promote the production and deposition of collagen and expressions of α-SMA in wounded tissues (Figures 1C, D). Both actions are aligned with wounds repair (Martin and Nunan, 2015). Furthermore, fibroblasts are essential effector cells for the progression of wound healing, and have been reported to participate in contractile force and collagen production (Martin and Nunan, 2015). Our results showed that corylin promoted migration, proliferation, and scratch healing of fibroblasts (Figures 3A–E). These effects may be associated with the activation of the PI3K/AKT pathway (Figures 3A–E). Corylin increased the phosphorylation of PI3K and AKT in fibroblasts (Figure 3E). Furthermore, inhibition of PI3K and AKT activities prevent the therapeutic effects of corylin (Figures 3A–D, 5A–C). Therefore, these data suggested that corylin may promote wounds repair by affecting the functions of fibroblasts.

Corylin is a flavonoid that is purified from *Psoralea coryliforlia*, and has been demonstrated to exhibit various biological properties through many signaling pathways, including SIRT1, NF-κB, PI3K/AKT and ERα signaling (Chen et al., 2020; Yu et al., 2020; Chen et al., 2021a; Chen et al., 2021b). Chen et al. (2021b) have demonstrated that corylin can directly bind with SIRT1 by forming a hydrogen bond with residue Gln345. Treatment with corylin in NIH/3T3-L1 adipocytes enhanced the SIRT1 activity (Chen et al., 2021b). In addition, our results showed that SIRT1 inhibitor EX527 could block the anti-inflammatory effects of corylin in RAW264.7 cells, further demonstrating the direct connection between the corylin and SIRT1. In the present study, we found that corylin promoted wounds repair through SIRT1/NF-κB and PI3K/AKT pathways, but not ERα signaling (Figures 4A–C). SIRT1/NF-κB signaling plays a key role in regulating inflammation and adhesion molecule expression in macrophages (Shen et al., 2021). Previous studies showed that corylin attenuates LPS-induced inflammatory responses in J-Blue cells through regulation of the NF-κB pathway (Hung et al., 2017). Consistently, our results suggested that corylin exhibited anti-inflammatory actions through inhibition of the acetylation of NF-κB p65 through SIRT1 (Figures 4A–C, 6A–C), indicating that the SIRT1/NF-κB signaling also plays an important role in wounds healing. Moreover, previous literature have provided important insight into the PI3K/AKT pathway in the functions and wounds healing (Castilho et al., 2013). Our data that corylin promotes wounds repair through activation of the PI3K/AKT pathway further supported this idea (Figures 5A–C). In addition, cory also affects the functions of the β3-adrenergic receptor, MAPKs, and IL-6/STAT3 signaling, future works need to be carried out to confirm the actions of these proteins.

It must be pointed out that one limitation of our study is that we have only used male mice in experiments. Further studies are necessary to fully characterize the potential effects of corylin in female animal. The other limitation is that we only studied the effects of corylin in cell line fibroblasts and macrophages. Future studies are necessary to purify primary fibroblasts and test the effects of corylin in these primary cells.

## 5 Conclusion

In summary, our results revealed that corylin promoted wounds healing through the PI3K/Akt pathway and SIRT1/NF-κB pathway. Our results also identify corylin as a promising therapy for chronic wounds healing.

## Data Availability

The original contributions presented in the study are included in the article/Supplementary Materials, further inquiries can be directed to the corresponding authors.

## References

[B1] Alam F. Khan G. N. Asad M. (2018). Psoralea corylifolia L: Ethnobotanical, biological, and chemical aspects: A review. Phytotherapy Res. PTR 32, 597–615. 10.1002/ptr.6006 PMC7167735 29243333

[B2] Castilho R. M. Squarize C. H. Gutkind J. S. (2013). Exploiting PI3K/mTOR signaling to accelerate epithelial wound healing. Oral Dis. 19, 551–558. 10.1111/odi.12070 23379329 PMC4764999

[B3] Che L. Yang H. Wang D. Liu S. (2021). Corylin sensitizes breast cancer cells to overcome tamoxifen resistance by regulating OAS1/miR-22-3p/SIRT1 axis. Acta Biochim. Pol. 68, 757–764. 10.18388/abp.2020_5663 34731560

[B4] Chen C. C. Kuo C. H. Leu Y. L. Wang S. H. (2021). Corylin reduces obesity and insulin resistance and promotes adipose tissue browning through SIRT-1 and β3-AR activation. Pharmacol. Res. official J. Italian Pharmacol. Soc. 164, 105291. 10.1016/j.phrs.2020.105291 33253817

[B5] Chen C. C. Li H. Y. Leu Y. L. Chen Y. J. Wang C. J. Wang S. H. (2020). Corylin inhibits vascular cell inflammation, proliferation and migration and reduces atherosclerosis in ApoE-deficient mice. Antioxidants (Basel) 9, 275. 10.3390/antiox9040275 32218307 PMC7222202

[B6] Chen I. C. Wang S. C. Chen Y. T. Tseng H. H. Liu P. L. Lin T. C. (2021). Corylin ameliorates LPS-induced acute lung injury via suppressing the MAPKs and IL-6/STAT3 signaling pathways. Pharm. (Basel) 14, 1046. 10.3390/ph14101046 PMC8537250 34681270

[B7] Dong J. Chen L. Zhang Y. Jayaswal N. Mezghani I. Zhang W. (2020). Mast cells in diabetes and diabetic wound healing. Adv. Ther. 37, 4519–4537. 10.1007/s12325-020-01499-4 32935286 PMC7547971

[B8] Dubey A. K. Podia M. Priyanka, Raut S. Singh S. Pinnaka A. K. (2021). Insight into the beneficial role of lactiplantibacillus plantarum supernatant against bacterial infections, oxidative stress, and wound healing in A549 cells and BALB/c mice. Front. Pharmacol. 12, 728614. 10.3389/fphar.2021.728614 34803678 PMC8600115

[B9] Han G. Ceilley R. (2017). Chronic wound healing: A review of current management and treatments. Adv. Ther. 34, 599–610. 10.1007/s12325-017-0478-y 28108895 PMC5350204

[B10] Hu Y. Lei S. Yan Z. Hu Z. Guo J. Guo H. (2021). Angelica dahurica regulated the polarization of macrophages and accelerated wound healing in diabetes: A network Pharmacology study and *in vivo* experimental validation. Front. Pharmacol. 12, 678713. 10.3389/fphar.2021.678713 34234674 PMC8256266

[B11] Hung Y. L. Fang S. H. Wang S. C. Cheng W. C. Liu P. L. Su C. C. (2017). Corylin protects LPS-induced sepsis and attenuates LPS-induced inflammatory response. Sci. Rep. 7, 46299. 10.1038/srep46299 28397806 PMC5387730

[B12] Kasuya A. Tokura Y. (2014). Attempts to accelerate wound healing. J. dermatological Sci. 76, 169–172. 10.1016/j.jdermsci.2014.11.001 25468357

[B13] Li H. Li X. Yang B. Su J. Cai S. Huang J. (2021). The retinoid X receptor alpha modulator K-80003 suppresses inflammatory and catabolic responses in a rat model of osteoarthritis. Sci. Rep. 11, 16956. 10.1038/s41598-021-96517-y 34417523 PMC8379249

[B14] Li Y. Zhou P. Hu T. Ren J. Xu Y. Qiu Y. (2021). NAAA inhibitor F96 attenuates BBB disruption and secondary injury after traumatic brain injury (TBI). Eur. J. Pharmacol. 912, 174561. 10.1016/j.ejphar.2021.174561 34655598

[B15] Liu Y. Liu Y. He W. Mu X. Wu X. Deng J. (2022). Fibroblasts: Immunomodulatory factors in refractory diabetic wound healing. Front. Immunol. 13, 918223. 10.3389/fimmu.2022.918223 35990622 PMC9391070

[B16] Martin P. Nunan R. (2015). Cellular and molecular mechanisms of repair in acute and chronic wound healing. Br. J. Dermatol 173, 370–378. 10.1111/bjd.13954 26175283 PMC4671308

[B17] Qin Y. Xie J. Zheng R. Li Y. Wang H. (2022). Oleoylethanolamide as a new therapeutic strategy to alleviate doxorubicin-induced cardiotoxicity. Front. Pharmacol. 13, 863322. 10.3389/fphar.2022.863322 35517792 PMC9065409

[B18] Sarojini H. Bajorek A. Wan R. Wang J. Zhang Q. Billeter A. T. (2021). Enhanced skin incisional wound healing with intracellular ATP delivery via macrophage proliferation and direct collagen production. Front. Pharmacol. 12, 594586. 10.3389/fphar.2021.594586 34220491 PMC8241909

[B19] Shen P. Deng X. Chen Z. Ba X. Qin K. Huang Y. (2021). SIRT1: A potential therapeutic target in autoimmune diseases. Front. Immunol. 12, 779177. 10.3389/fimmu.2021.779177 34887866 PMC8650132

[B20] Singh T. P. Zhang H. H. Borek I. Wolf P. Hedrick M. N. Singh S. P. (2016). Monocyte-derived inflammatory Langerhans cells and dermal dendritic cells mediate psoriasis-like inflammation. Nat. Commun. 7, 13581. 10.1038/ncomms13581 27982014 PMC5171657

[B21] Takeo M. Lee W. Ito M. (2015). Wound healing and skin regeneration. Cold Spring Harb. Perspect. Med. 5, a023267. 10.1101/cshperspect.a023267 25561722 PMC4292081

[B22] Tardaguila-Garcia A. Garcia-Morales E. Garcia-Alamino J. M. Alvaro-Afonso F. J. Molines-Barroso R. J. Lazaro-Martinez J. L. (2019). Metalloproteinases in chronic and acute wounds: A systematic review and meta-analysis. Wound Repair Regen. 27, 415–420. 10.1111/wrr.12717 30873727

[B23] Wang Y. Feng Z. Yang M. Zeng L. Qi B. Yin S. (2021). Discovery of a novel short peptide with efficacy in accelerating the healing of skin wounds. official J. Italian Pharmacol. Soc. 163, 105296. 10.1016/j.phrs.2020.105296 33220421

[B24] Williams D. L. Gonzalez Villavincencio C. M. Wilson S. (2006). Chronic ocular lesions in tawny owls (*Strix aluco*) injured by road traffic. Vet. Rec. 159, 148–153. 10.1136/vr.159.5.148 16877681

[B25] Wu K. Xiu Y. Zhou P. Qiu Y. Li Y. (2019). A new use for an old drug: Carmofur attenuates lipopolysaccharide (LPS)-Induced acute lung injury via inhibition of FAAH and NAAA activities. Front. Pharmacol. 10, 818. 10.3389/fphar.2019.00818 31379583 PMC6659393

[B26] Xie X. Li Y. Xu S. Zhou P. Yang L. Xu Y. (2022). Genetic blockade of NAAA cell-specifically regulates fatty acid ethanolamides (FAEs) metabolism and inflammatory responses. Front. Pharmacol. 12, 817603. 10.3389/fphar.2021.817603 35069223 PMC8777083

[B27] Yu A. X. Xu M. L. Yao P. Kwan K. K. Liu Y. X. Duan R. (2020). Corylin, a flavonoid derived from Psoralea Fructus, induces osteoblastic differentiation via estrogen and Wnt/β-catenin signaling pathways. FASEB J. 34, 4311–4328. 10.1096/fj.201902319RRR 31965654

[B28] Zheng Z. G. Zhang X. Liu X. X. Jin X. X. Dai L. Cheng H. M. (2019). Inhibition of HSP90β improves lipid disorders by promoting mature SREBPs degradation via the ubiquitin-proteasome system. Theranostics 9, 5769–5783. 10.7150/thno.36505 31534518 PMC6735373

[B29] Zhou P. Xiang L. Yang Y. Wu Y. Hu T. Liu X. (2019). N-Acylethanolamine acid amidase (NAAA) inhibitor F215 as a novel therapeutic agent for osteoarthritis. Pharmacol. Res. official J. Italian Pharmacol. Soc. 145, 104264. 10.1016/j.phrs.2019.104264 31063807

