# Supplementary material for: Corylin accelerated wound healing through SIRT1 and PI3K/AKT signaling: a candidate remedy for chronic non-healing wounds
Source: Front Pharmacol. 2023 May 17;14:1153810. doi: 10.3389/fphar.2023.1153810 (PMC10229780; doi:10.3389/fphar.2023.1153810)
Supplement: Supplementary file 1 [file DataSheet1.docx]

Corylin accelerated wound healing through SIRT1 and PI3K/ AKT signaling: a candidate remedy for chronic non-healing wounds

Yanghui Xiu et. al.


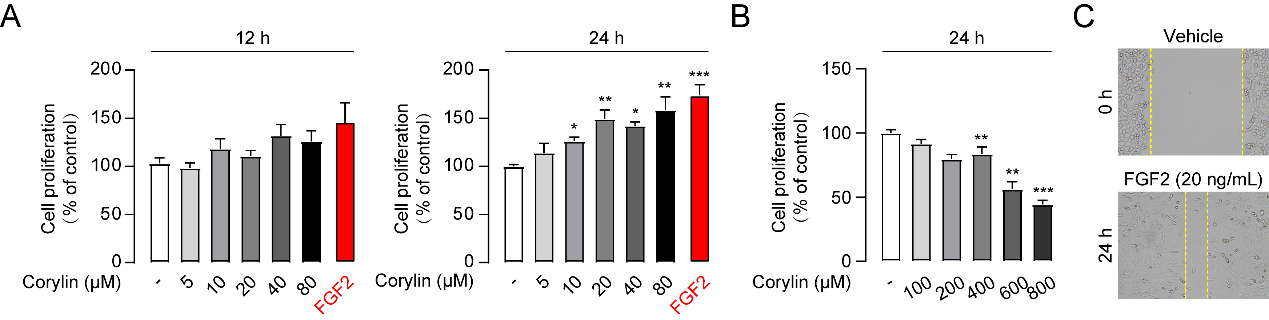


**Figure S1. The effects of corylin on cell proliferation**. (A) Low doses and (B) high doses of corylin and FGF2 (20 ng/mL) affected NIH/3T3 cell proliferation at 12 h and 24 h after treatment. N = 3, *, P < 0.05; **, P < 0.01; ***, P < 0.001 vs control.


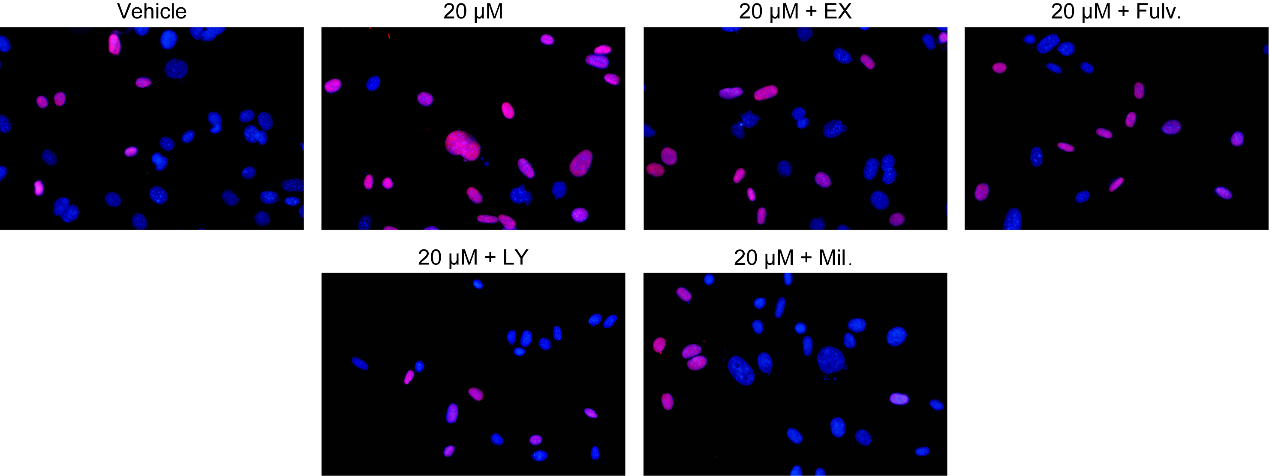


**Figure S2. Corylin facilitated the proliferation of NIH/ 3T3 cells.** EdU assay of corylin on the NIH/3T3 cells proliferation.
